# Ovariotomy — Clinic of Prof. Gross

**Published:** 1873-11

**Authors:** 


Ovariotomy.—*Clinic of Prof. S. D. Gross, M. D.*—This woman, 30 years of age, has suffered for nearly four years with a cystic tumor of the ovary, which has been tapped twice within the past six months, twenty-six quarts of fluid being drawn off at the first operation, and nineteen quarts at the second. The fluid has re-accumulated in large quantity since the last paracentesis, and she now comes to the clinic to have the tumor removed by ovariotomy.

It is important to see that the bowels and bladder of the patient are emptied, and that the room is kept well warmed during the operation. An incision two and a half inches in length is then made through the linea alba, extending from the pubes upwards, which opening, if the adhesions are not extensive, will be sufficiently large to admit of the removal of the tumor, especially as its contents will be previously drawn off by the trocar. After the superficial incision has been made, a probepointed bistoury is introduced, and the muscular wall of the abdomen laid open, exposing the cyst to view, which proves to be multilocular, and when punctured gives a thick yellowish fluid of the consistency of soft soap.

I now seize the sac and endeavor to draw it out through the incision, rupturing with the finger or dividing with the knife the adhesions holding it to the adjoining viscera. It is firmly adherent to the omentum; and, as the attachment is very vascular, a ligature is placed around it before it is cut.

After the cyst is drawn through the opening, the pedicle by which the tumor is attached to the ovary is divided, having been surrounded by a clamp, in order that there may be no possibility of hemorrhage. After all the blood that has fallen into the abdominal and pelvic cavities has been thoroughly removed by perfectly clean sponges, lest peritonitis or pyæmia be induced, the stump of the pedicle, embraced by the clamp, is brought out by the lower extremity of the incision. The edges of the wound are then brought together by several points of interrupted suture, and four pins passing through the muscles and parietal peritoneum. It is necessary to leave an opening at the lower part of the wound, to insure drainage of the effused fluids, the retention of which might induce septicæmia.

An oiled compress is placed over the wound and held in position by long strips of adhesive plaster, after which a mass of cotton is laid on the abdomen and the patient's body surrounded by a broad bandage of flannel. The bowels will be locked up by opium, and the woman kept perfectly at rest, in a room well warmed and free from draughts of air, with her legs flexed on the pelvis, to relax the muscles and thus prevent tension upon the contents of the abdomen. If effusions of a serous or purulent nature occur, the pelvic cavity must be washed out with tepid water and chlorinated soda or permanganate of potassa by means of a syringe, lest the accumulation of these fluids within the pelvis set up inflammation or induce pyæmia.

